# A pilot of the feasibility and usefulness of an aged obese model for use in stroke research

**DOI:** 10.12688/wellcomeopenres.16592.1

**Published:** 2021-05-10

**Authors:** Annastazia E. Learoyd, Ryan Calmus, Chelsea N. Cunningham, Tim J. England, Tracy D. Farr, Kevin C.F. Fone, David A. Kendall, Saoirse E. O’Sullivan, Rebecca C. Trueman

**Affiliations:** 1 School of Life Science, University of Nottingham, Nottingham, NG7 2UH, UK; 2 Newcastle University Biosciences Institute, Newcastle University, Newcastle, NE2 4HH, UK; 3 Division of Medical Sciences & GEM, School of Medicine, University of Nottingham, Nottingham, DE22 3DT, UK; 4 University Hospitals of Derby and Burton, NHS trust, Derby, DE22 3NE, UK; 5 PharmNovo AB, 2 Woodlands Lane, Wirral, CH48 8DA, UK

**Keywords:** Stroke, Animal models of Stroke, Obesity, Ageing, hypertension, co-morbidities

## Abstract

**Background:** Animal models of stroke have been criticised as having poor predictive validity, lacking risk factors prevalent in an aging population. This pilot study examined the development of comorbidities in a combined aged and high-fat diet model, and then examined the feasibility of modelling stroke in such rats.

**Methods:** Twelve-month old male Wistar-Han rats (n=15) were fed a 60% fat diet for 8 months during which monthly serial blood samples were taken to assess the development of metabolic syndrome and pro-inflammatory markers. Following this, to pilot the suitability of these rats for undergoing surgical models of stroke, they underwent 30min of middle cerebral artery occlusion (MCAO) alongside younger controls fed a standard diet (n=10). Survival, weight and functional outcome were monitored, and blood vessels and tissues collected for analysis.

**Results:** A high fat diet in aged rats led to substantial obesity. These rats did not develop type 2 diabetes or hypertension. There was thickening of the thoracic arterial wall and vacuole formation in the liver; but of the cytokines examined changes were not seen. MCAO surgery and behavioural assessment was possible in this model (with some caveats discussed in manuscript).

**Conclusions:** This study shows MCAO is possible in aged, obese rats. However, this model is not ideal for recapitulating the complex comorbidities commonly seen in stroke patients.

## Introduction

Stroke most commonly occurs in people over the age of 65 years and this population often has several additional comorbidities such as hypertension, diabetes or cholesterolemia ^1^. Animal models of stroke are most commonly produced using young, healthy rodents and rarely include comorbidities. This has been suggested as a contributory factor in the failure of translation of new stroke treatments ^2, 3^.

A substantial body of preclinical stroke research has been conducted in hypertensive and diabetic animals ^4^. However, the mechanism for inducing co-morbidities in many of these models is very specific and artificial compared to the human condition. There is a renewed interest in combining additional comorbidities, such as age and obesity. Age is the most important risk factor for stroke ^5, 6^, and since the publication of the STAIR (stroke therapy academic industry roundtable) criteria ^7^ it is becoming more common to see this incorporated into preclinical stroke models. As one might expect, several studies have reported worse outcomes after stroke in aged animals; reviewed in 8. Interestingly, some studies have reported the opposite in that there was a decrease in infarct volumes in aged stroke animals accompanied by attenuation of the brain inflammatory responses to stroke ^9^.

Inducing obesity in rodents can be accomplished via provision of a high fat diet which has been shown to increase body fat, blood pressure and leptin and insulin levels in the blood ^10^, reviewed in 11. Under these circumstances in young adult animals, outcome after stroke has been reported to be poorer ^12, 13^. In young mice, a high fat diet increased blood glucose and cerebral microvascular density and resulted in larger infarcts, oedema, haemorrhagic transformation and greater loss of neurological function, potentially via increases in matrix metalloproteinase 9 in the ischemic brains ^14^. Another study, which fed young adult mice a high-fat diet, showed that outcome was dependent on the duration for which the high fat diet was provided (worse outcome with longer periods), and while there were no changes in blood pressure, obese mice exhibited higher levels of inflammatory markers in the brain ^15^.

Inflammation is closely associated with aging, obesity and stroke ^9, 12, 15^, but few studies have looked at the combination of these factors. Obesity induced by a high fat diet, starting at a young age has been examined in rodents, for example ^15– 17^. None of these previous studies examined animals older than 14 months (senescence is considered to be above 18 months in mice ^18^), or started a high fat diet in adulthood. Here, we have piloted the use of a high fat diet in aged rats (12–20 months) to mimic a poor diet in adulthood and characterised the development of markers of comorbidities and the utility of the model for stroke research. This was with the view to create a model with a range of comorbidities for assessment of therapeutics for stroke. This experiment was an initial pilot, which performed a within animal comparison of metabolic / inflammatory markers from baseline (aged 12 months) to 8 months on a high fat diet. This was to establish if a robust phenotype would develop in these animals by introducing a high fat diet, the attrition rates, and to practically assess the feasibility of performing behavioural tests and stroke surgical procedures on these rats with the purpose to inform the design of larger future studies.

## Methods

### Ethical approval

All experiments were performed under a project license (40/3682) approved by the UK Home Office and University of Nottingham, UK, Animal Welfare and Ethical Review Body, and were carried out in accordance with EU and UK guidelines and regulations. All efforts were made to ameliorate harm to the animals. This included regular welfare checks throughout the study (daily checks, with increased frequency following surgery (more than 4x per day)), medication including pain relief and saline injection as necessary, and careful consideration of the procedures undertaken, the methods used and their necessity. 

### Animals and experimental design

Male Wister-Han rats aged 12 months (Charles River, France, 500-650g, n=15) were housed in groups of 3 under standard conditions (individually ventilated cages (IVC), 21-23°C, 12h light-dark cycle) with unlimited access to food and water. They were provided with a 60% high fat diet (D12492, Research Diets Inc., US) for 8 months. During this time, blood samples were withdrawn to assess potential changes in metabolic and inflammatory markers across the time course of the diet intervention (described in detail below). Three rats were euthanised within 8 months because of tumours. Euthanasia was completed following Schedule 1 of the UK Animals (Scientific Procedures) Act 1986. The method used was an intraperitoneal injection of Euthatal followed by dislocation of the neck and decapitation as a confirmation method.

**Figure 1.**
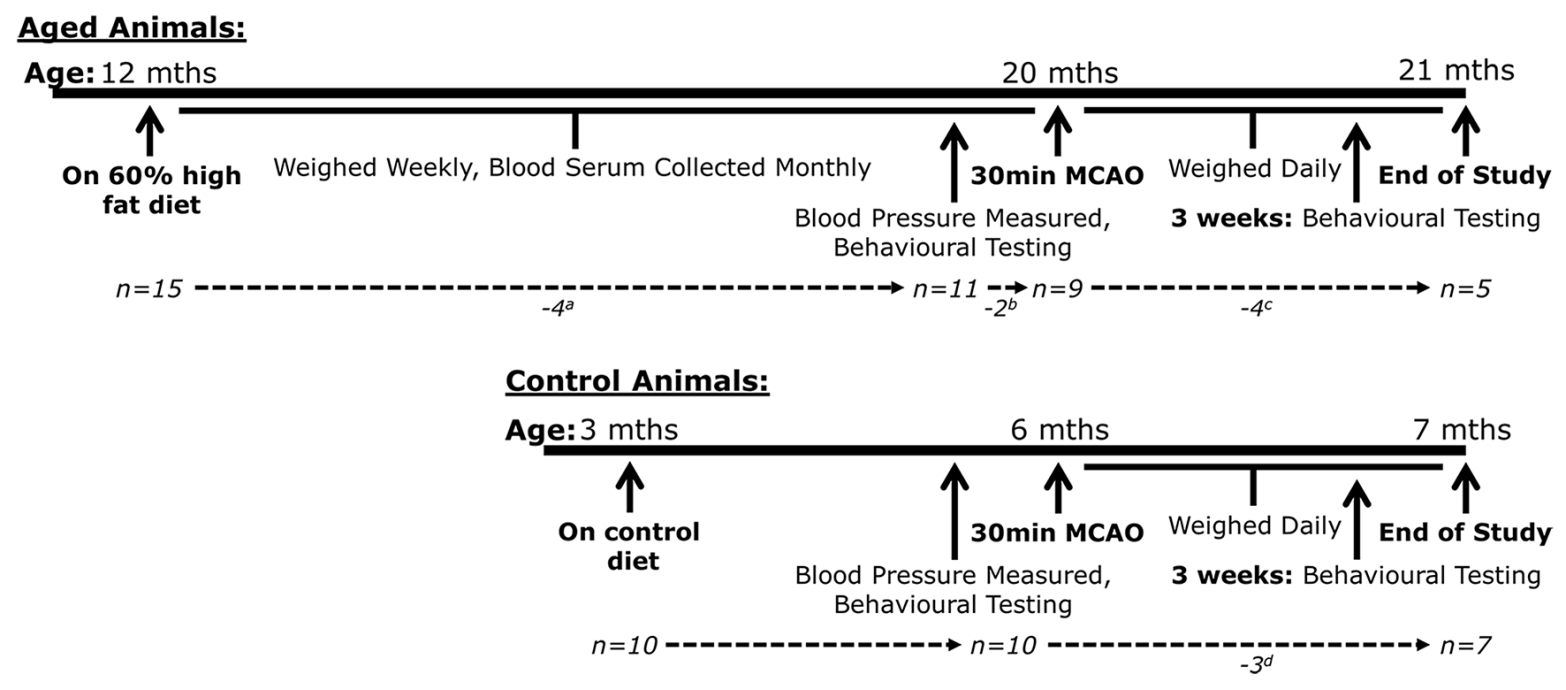
Timeline of Procedures Undertaken by Aged and Control Animals Across the Study. Male Wister-Han rats were fed a high fat diet containing 60% fat for 8 months before undergoing a 30 minute middle cerebral artery occlusion (MCAO) alongside younger rats fed a control diet. Aged animals were assessed regularly for the development of comorbidities over the initial 8 months. Both aged and control animals were tested for behavioural deficits prior to and 3 weeks after MCAO surgery. Animals were euthanised 4 weeks after MCAO surgery. The numbers of animals is shown underneath each timeline. ^a^ 3 aged animals were euthanised in the first 8 months due to tumours, while another was excluded from all previous analysis due to the discovery of a tooth abscess (n=1). Before its exclusion the animal was used to pilot MCAO occlusion length, along with 2 other aged animals ^b^ and then euthanised 24 hours later. ^c^ 4 aged animals were removed from the study post-MCAO. One suffered from pulmonary oedema at 24hrs. The other 3 were euthanised by 16 days post-MCAO due to dermal burns from the homoeothermic heating system during surgery. This was an unforeseen consequence of the large subcutaneous regions of adipose tissue in these rats that did not have a sufficient blood supply to disperse heat provided by a homoeothermic blanket during surgery. Monitoring of both internal and dermal temperature successfully counteracted this issue. ^d^ 3 control animals were removed from the study post-MCAO. One was euthanised due to an oesophageal compaction. The other 2 did not exhibit evidence of infarction.

Rats which reached 20 months of age (n=12) had blood pressure measurement collected and then were used to confirm the feasibility of middle cerebral artery occlusion surgery (MCAO) and to evaluate typical behavioural tests for use with obese rats. Three rats were used to pilot MCAO occlusion length (for 20 min and 30 min) as required by ethical approval, one of which had developed a tooth abscess and was subsequently excluded from blood analyses. The remaining rats (n=9) underwent 30min of MCAO. All rats underwent behavioural testing prior to and at 3 weeks post-surgery. A protocol timeline is depicted in Figure 1. Exclusions were applied after MCAO surgery, details of which are shown in Table 1.

**Table 1.** Animal exclusions for each analysis. A number of animals had to be excluded of the course of the study due to health concerns, adverse events or a lack of infarction. This has led to varying numbers for each analysis with the exclusions for each analysis described here.

Outcome measure	Group	Number of rats in analysis	Number excluded	Reason for exclusion
***Diet induced changes***				
**Weight gain**	Aged	11	3 1	Tumours Tooth Abscess
**Metabolic alterations**	Aged	11	3 1	Tumours Tooth Abscess
	Control	9-10	(9 samples randomly selected for ELISAs)	
**Inflammatory changes**	Aged	11	3 1	Tumours Tooth Abscess
**Liver and vessel pathology**	Aged	5	3 1 2 1 3	Tumours Tooth Abscess Piloting MCAO surgery Pulmonary oedema Heating Issues
	Control	9	1	Oesophageal compaction
***Post-MCAO measures***				
**Weight loss**	Aged	5	3 1 2 1 3	Tumours Tooth Abscess Piloting MCAO surgery Pulmonary oedema Heating Issues
	Control	7	1 2	Oesophageal compaction No infarction
**Infarct volume**	Aged	5	3 1 2 1 3	Tumours Tooth Abscess Piloting MCAO surgery Pulmonary oedema Heating Issues
	Control	7	1 2	Oesophageal compaction No infarction
**Behavioural deficits (pre-MCAO)**	Aged	9	3 1 2	Tumours Tooth Abscess Piloting MCAO surgery
	Control	10		
**Behavioural deficits (post-** **MCAO)**	Aged	5	3 1 2 1 3	Tumours Tooth Abscess Piloting MCAO surgery Pulmonary oedema Heating Issues
	Control	7	1 2	Oesophageal compaction No infarction

Additionally, an adult control group was included (Charles River, France, n=10, 250–350g, 3 months of age on arrival and received control diet (D12450J, Research Diets Inc., US) for a further 3 months to provide a bench mark for behavioural performance, MCAO surgery, blood pressure, metabolic measures and terminal histological measures.

Randomisation was not possible, as the animals were purchased as aged or young and within the groups, they underwent the same manipulations (diet and MCAO surgery). Blinding was also not possible during blood sampling, surgery and behaviour as there was an obvious size difference between animals. Histological images were blinded for analysis.

The initial number of rats was selected as appropriate for a longitudinal feasibility study, as if a robust phenotype was not evident with 12 animals it would limit the feasibility of using this model for stroke research. Stroke models are inherently variable and adding to this a variable or very subtle co-morbidity model would require unfeasibly large group sizes to achieve appropriate power. All procedures were completed during daylight hours (8am-6pm). Animal welfare was monitored weekly prior to MCAO and multiple times a day for 48hrs after surgery, then at least daily.

### Blood sampling and analysis

To assess changes in metabolic and inflammatory markers over the time course of the diet intervention, local anaesthetic was applied to the tail (5% EMLA cream, AstraZeneca, UK) and rats were placed in a heated box at 37 °C for 5min. They were restrained with a towel, and 1mL of blood was collected from the lateral tail vein. A drop of blood was collected in a microcuvette to determine glucose concentration (HemoCue Glucose 2.1 ^+^). Blood was collected in tubes with serum separating gel (Greiner Bio-one, vol.: 1.3ml) and left to clot for 15–30 minutes at room temperature before being centrifuged at 1,000xg for 10 minutes. The serum was aliquoted and analysed for various metabolic and inflammatory markers using bead-based Multiplex assays (RMHMAG-84K and RECYTMAG-65K, Merck Millipore, Germany) and measured on a MAGPIX system (Merck Millipore, Germany). Preliminary analysis of 23 different inflammatory markers was carried out on serum from the 8-month time point, which lead to 11 markers being selected for the full-time course analysis. Blood samples for biomarker analysis were taken once a month. A small number of samples (metabolic: 13/90, inflammatory: 11/90) were excluded from biomarker analysis due to an insufficient quantity of high-quality blood serum. Random glucose tests were performed once a month, and from day 98 on the high fat diet fasted glucose tests (food removed for 20hrs) were also performed monthly.

### Blood pressure measurement

Following 8 months on the high fat diet, rats were habituated (20min/day for 3 days) to restraint on a heated pad (CODA High Throughput Non-invasive Blood Pressure system with 4.1 software, Kent Scientific, US). Tail cuffs and a one volume pressure recording sensor were placed as close to the base of the tail as possible. Monitoring lasted for 10 minutes and consisted of 15 inflations.

### Middle cerebral artery occlusion

A stroke was induced using the filament model as described previously ^19– 21^. Briefly, rats were anaesthetised using isoflurane in a 70:30 nitrous oxide: oxygen mixture. Body temperature was maintained at 37°C using a homeothermic system (Harvard Apparatus, US). A silicone-coated nylon filament (diameter: 0.39–0.41mm, Doccol, USA) was advanced up the right common carotid, into the internal carotid artery, until a decrease in blood flow was observed with a Laser Doppler probe (Moor Instruments, UK) against the temporal bone. Rats were recovered, functional deficits noted, and briefly re-anaesthetised after 30 minutes to remove the filament and ligate the common carotid. Local anaesthetic was applied to the surgical site and rats were given 1mg/ml paracetamol in the drinking water from 12h prior to until 48h after surgery along with mashed diet. Subcutaneous injections of saline (5mg/kg) and atropine (0.1mg/kg, Animalcare Ltd., UK) were given at the time of surgery. This procedure was completed during daylight hours from 8am until 5pm.

### Behavioural analysis

The feasibility of testing behaviour using the Bilateral Asymmetry, Cylinder, and Stepping tests in aged obese rats was assessed. It was not possible to perform blinded behavioural testing as the aged rats were significantly bigger. Bilateral Asymmetry involved the random placement of sticky squares (7×7mm) onto the inside of both forepaws. The time taken to touch and remove was measured four times. The order of testing of paws was randomised for each animal. The Cylinder test involved placing rats in a Perspex cylinder (37.5cm tall and 25cm diameter) for 5 minutes. An observer scored the video for paw use for the first 20 touches. However, this test had to be abandoned due to the inactivity of the aged rats. The Stepping test was performed by holding rats at a 45° angle so that one forepaw was resting on a table while the animal was moved laterally for 1 metre. The number of adjustment steps was counted. The test was randomly repeated 3 times with both forepaws in forehand and backhand directions. For detailed protocols see Trueman *et al.* ^21^. These procedures were completed during daylight hours from 8am until 5pm.

### Tissue collection, histology and analysis

Four weeks after MCAO, rats were deeply anaesthetised and exsanguinated, to allow collection of blood. Death was confirmed by dislocation of the neck and decapitation. The brains, livers and arteries (thoracic aorta and femoral) were collected. Brains were snap frozen, stored at -80°C, embedded in Optimum temperature cutting compound (OCT, Cell Path, UK), and sectioned at 20µm on a cryostat (HM 505E, Microm, UK) onto 3-Aminopropyltriethoxysilane (APES) coated slides. Every 24 ^th^ section was post-fixed with 4% paraformaldehyde (PFA) for 5min, stained with Cresyl Violet and photographed (DS3100, Nikon, UK) to determine infarct volume. ImageJ software (NIH, US) was used to manually delineate infarcted tissue and measurements were summated and multiplied by slice thickness and the number of sections in a series to give the infarct volume.

Livers and arteries were post-fixed for 24 hours in 4% PFA, embedded in paraffin and sectioned at 20µm on a microtome (Cut 4060, Slee Mainz, Germany) into a 60°C water bath. Arterial segments were stained with Hematoxylin and Eosin. Sections were photographed using a microscope (Axioplan, Zeiss, Germany. x10) and arterial wall thickness measured with ImageJ. Liver sections were stained with 0.35% Oil Red O for 50min, and de-stained in 78% methanol until the background was clear. The number of fatty vacuoles in the liver was counted manually. All tissue analysis was completed while blinded to animal group.

### Exclusions

Prior to MCAO animals were excluded from the blood sample analyses if they developed health issues during the study, as these could have confounded the measures. This included an abscess and tumours. Following MCAO rats were excluded due to adverse events and incomplete MCAO resulting in no infarct; full details are in Table 1. These criteria were decided *a priori*. In addition, blood samples were excluded if they were of insufficient quantity or quality to analyse.

### Statistical analysis

Data are presented as means ± standard deviation (SD), the experimental unit was the rat. GraphPad Prism 8 was used to analyse weight gain across the time course (using a repeated measures one-way analysis of variance (ANOVA) with Greenhouse-Geisser correction), monthly weight gain (using a Kruskal-Wallis test), metabolic, and inflammatory markers (using a matched mixed-effects model with restricted maximum likelihood and Greenhouse-Geisser correction, to account for missing values) assessing changes across the time course within the aged high fat diet group. Benchmark values from the adult rats on standard diet are included on the graphs for information purposes. Pre-stroke behaviour was compared between aged and adult control groups using a two-way ANOVA (group and side of body) and blood pressure was compared using unpaired Student’s t tests. SPSS, version 24 was used to analyse weight loss after surgery using a two-way repeated measures ANOVA (Time and Group). *Post-hoc* Bonferroni multiple comparison corrections were used and p<0.05 considered significant. All data was assessed for normality prior to statistical analysis using Q-Q plots. Only monthly weight gain and the results from the Bilateral Asymmetry test did not match a normal distribution. Post-stroke data (infarct volume, histological measures and behaviour) are presented descriptively and not statistically analysed, as the study was not designed or powered for these comparisons. All statistical tests are standard and could be carried out in an open-access statistical software package, such as R.

## Results

### A high fat diet induced weight gain but no significant metabolic alterations in aged animals

**Figure 2.**
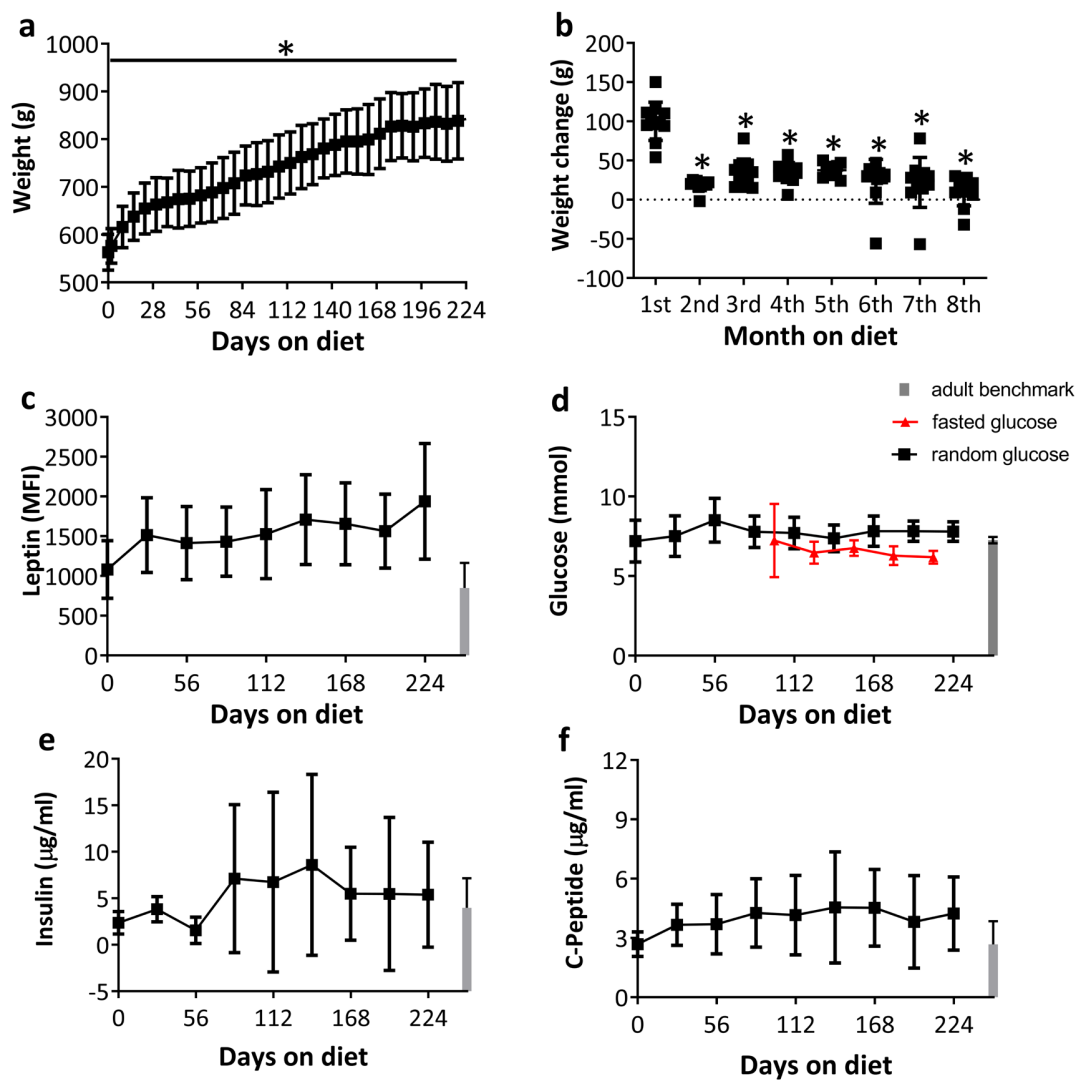
Changes in weight and metabolic markers in aged rats fed a high fat diet (n=11) over 8 months. **a**: Aged rats gained a significant amount of weight over the 8 months (p<0.001). **b**: Most of the weight was gained in the first month on the diet (p<0.001). **c**: Serum leptin levels increased over the 8 months, albeit this increase did not reach significance (p=0.058). Blood serum concentrations of glucose random (black), fasting (red) ( **d**), Insulin ( **e**) and C-peptide ( **f**) did not significantly change over the 8 months. * p<0.05 compared to baseline measures. Adult normal diet controls presented in grey bars as benchmark (n = 9-11). Data are presented as means±SD.

Consumption of the high fat diet caused a dramatic increase in weight ( Figure 2A, F _2.1,21.5_=142.70, p<0.001) that was apparent as early as 2 days after diet initiation (p<0.05) ^22^. Interestingly, the majority of the weight was acquired in the first month ( Figure 2B), after which, gain remained relatively consistent (KW=49.18, p<0.001). Serum leptin levels followed the same pattern but the increase in leptin concentration did not quite reach significance ( Figure 2C, F _2.7,22.4_=2.99, p=0.058).

Blood glucose did not alter over the 224 days ( Figure 2D, F _4.4,44.0_=1.63, p=0.18). Insulin serum levels tended to increase and were more variable between animals. This was not significant compared to baseline ( Figure 2E, F _2.7,22.7_=1.52, p=0.24). Fasted glucose levels were measured from 98 to 210 days, no change was seen over this period (F _1.2,11.7_ =1.87, p=0.20). There were no changes in C-peptide over time ( Figure 2F, F _2.9,24.4_=1.44, p=0.26).

### Aged animals did not exhibit changes in inflammatory markers in response to a high fat diet

**Figure 3.**
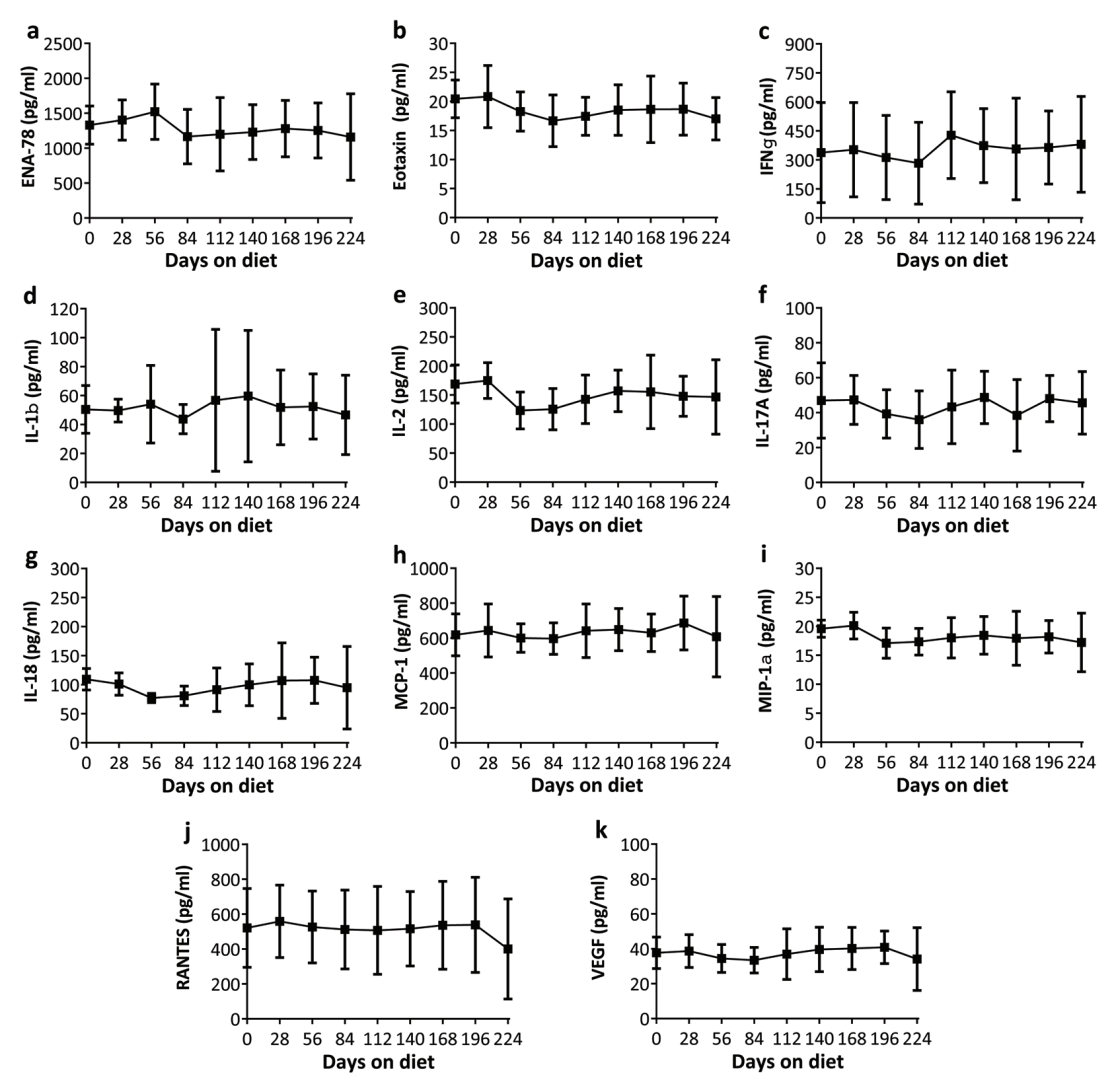
Changes in serum inflammatory markers in aged rats with a high fat diet over 8 months (n=8-11/timepoint – due to missing values). None exhibited significant changes. **a**– **k**: ENA-78 ( **a**, p=0.27), eotaxin ( **b**, p=0.29), IFN-γ ( **c**, p=0.29), IL-1β ( **d**, p=0.50), IL-2 ( **e**, p=0.22), IL-17A ( **f**, p=0.39), IL-18 ( **g**, p=0.30), MCP-1 ( **h**, p=0.48), MIP-1α ( **i**, p=0.26), RANTES ( **j**, p=0.12), VEGF ( **k**, p=0.33). Data are presented as means±SD.

Eleven cytokines were selected for analysis over the course of the diet. None were found to exhibit any significant changes in concentration. The cytokines analysed were epithelial-derived neutrophil-activating peptide 78 (ENA-78, Figure 3A, F _3.3,28.1_=1.39, p=0.27), eotaxin ( Figure 3B, F _3.1,26.9_=1.32, p=0.29), interferon-γ (IFN-γ, Figure 3C, F _0.8,6.6_=1.32, p=0.29), interleukin-1β (Il-1β, Figure 3D, F _1.7,14.6_=0.66, p=0.50), interleukin-2 ( Figure 3E, F _3.1,26.9_=1.57, p=0.22), interleukin-17A ( Figure 3F, F _3.5,29.8_=1.05, p=0.39), interleukin-18 ( Figure 3G, F _1.3,11.2_=1.25, p=0.30), monocyte chemoattractant protein-1 (MCP-1, Figure 3H, F _1.7,14.9_=0.73, p=0.48), macrophage inflammatory protein-1α (MIP-1α, Figure 3I, F _2.4,20.6_=1.43, p=0.26), regulated on activation, normal T expressed and secreted protein (RANTES, Figure 3J, F _3.4,29.4_=1.32, p=0.12) and vascular endothelial growth factor (VEGF, Figure 3K, F _3.3,28.0_=1.20, p=0.33).

### Age in combination with the high fat diet resulted in liver and vessel pathology

**Figure 4.**
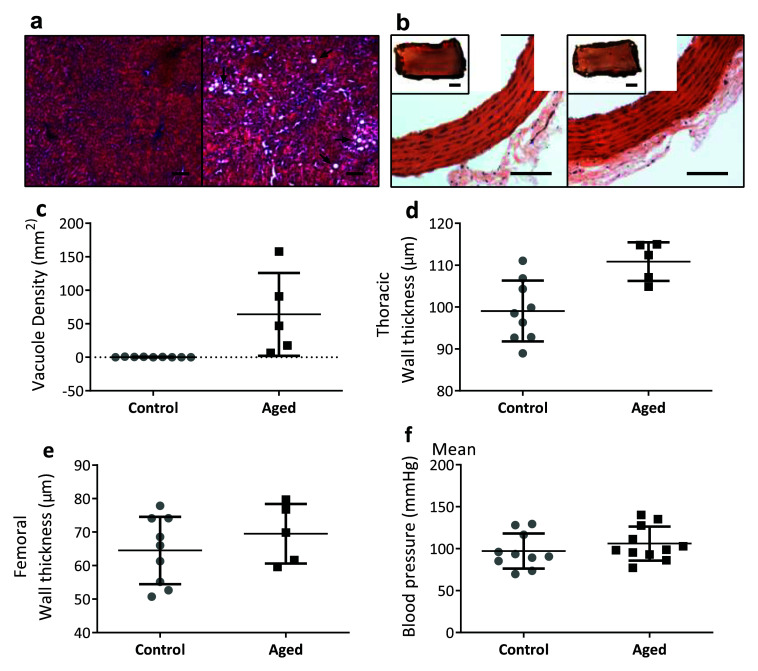
Liver and arterial wall pathology in response to a high fat diet. **a**: Representative images from the livers of a control (left) and an aged/obese rat (right) (scale bar indicates 100µm). **b**: Representative images of thoracic arteries from a control (left) and aged/obese rat (right). Inset: images of fat deposition with Oil Red O (all scale bars indicate 100µm). **c**: Increased fat vacuoles (white areas and arrows in a) were observed in aged/obese rats (n=5) compared to controls (n=9) (p=0.008). **d**– **e**: Thoracic arteries ( **d**) in aged/obese rats were thicker than in controls, however femoral arteries were not ( **e**). **f**: No difference was seen in blood pressure (control n = 10, aged = 11). Data are presented as means±SD.

There was evidence of fat accumulation in the liver in aged rats on a high fat diet compared to control rats ( Figure 4A and C). Interestingly, there were no significant differences in blood pressure between aged rats following 8 months on a high fat diet (106.0±20.3mmHg) compared to young rats on a normal diet (97.2±20.9mmHg) ( Figure 4F, t _19_=0.98, p=0.34). This was despite evidence suggesting thickness in the thoracic arteries in aged rats on a high fat diet increased ( Figure 4B and D). With the limited data available no obvious differences were evident between femoral arteries of the two groups ( Figure 4E). A qualitative analysis of longitudinal artery slices stained with Oil Red O showed no increased fat deposition in aged rats with a high fat diet (inserts Figure 4B).

### Age and obesity resulted in increased weight loss post-stroke with no greater effect on functional deficits

**Figure 5.**
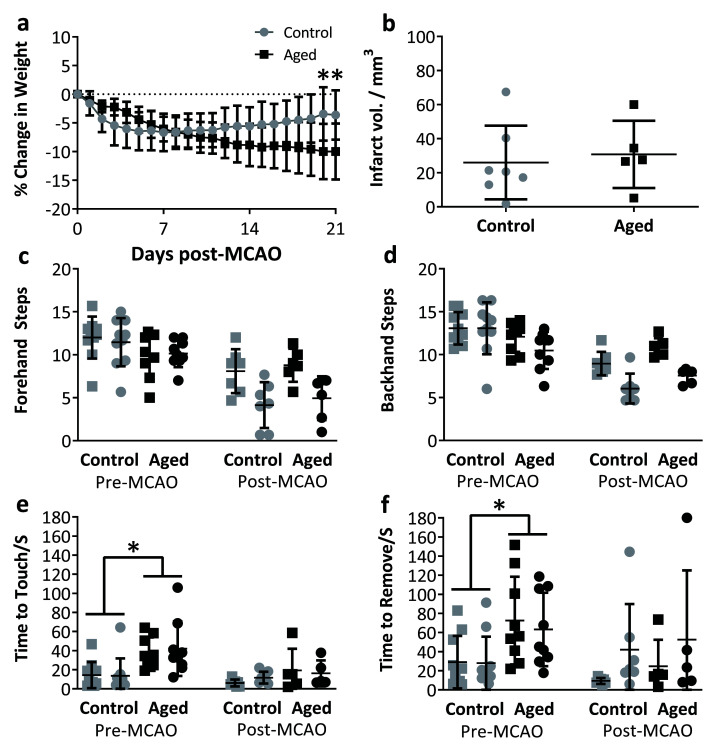
Morbidity and functional outcomes after MCAO in aged rats fed a high fat diet for 8 months compared to adult rats on a regular diet **a**: Aged rats on the high fat diet continued to lose weight in the 3 post-operative weeks while control rats began to recover weight after the first week (p<0.001). **b**: Infarct volume in aged/high fat and control rats. **c**– **d**: Adjusting steps test, square symbol: ipsilateral paw, circle: contralateral paw, no significant difference in number of steps was seen between control and aged animals prior to MCAO. **e**– **f**: Adhesive removal, square symbol: ipsilateral paw, circle: contralateral paw, aged animals took longer to contact and remove the adhesive prior to MCAO surgery (p=0.0025 & p=0.015). Pre-MCAO n=(control = 10, aged = 11), post-MCAO n=(control = 8, aged = 5). Data are presented as means±SD. Post-MCAO behavioural data presented for descriptive purposes.

MCAO surgery was successful in these larger aged and obese rats. There was a reduction in body weight following surgery in all rats ( Figure 5A, F _21,210_=18.33, p<0.001) that did not significantly differ between groups (F _1,10_=0.88, p=0.37). However, there was a significant interaction between time and group (F _21,210_=10.23, p<0.001); aged rats on a high fat diet continued to lose weight until 21 days post-surgery, whereas the rats on control diet began gaining weight after the first post-operative week ( Figure 5A). From the limited data available, there was no difference in infarct volumes between groups ( Figure 5B).

Prior to MCAO, control and aged rats did not exhibit differences in performance on the stepping task ( Figure 5: forehand: Animal Group: F _1, 17_ =2.61, p=0.124; backhand: Group: F _1,17_=3.57, p=0.076). However on the bilateral asymmetry task, aged rats on the high fat diet took significantly longer to contact and remove stimuli on both paws in comparison to young adult rats prior to surgery ( Figure 5, Group: Contact: F _1,17_ =12.53, p=0.0025, Remove: F _1, 17_ =7.245, p=0.015)

After MCAO, both aged and young adult rats were able to perform the tasks ( Figure 5), but due to low numbers are presented descriptively and not statistically analysed. There is some evidence on the stepping task for a reduction in adjusting steps with the contralateral paw in both the forehand and backhand directions. The data from adhesive removal is too variable to draw conclusions based on the small number presented.

## Discussion

The aim of this study was to develop a rat model that contained multiple comorbidities (including age) for use in stroke research. Aged rats fed a high fat diet did not induce a robust range of inflammatory, metabolic and physiological changes. Stroke surgery and behavioural assessment were feasible in these rats, with some consideration.

A review of studies that made use of a high fat diet (typically in young animals) documented several physiological changes with the following being most common ^11^: weight gain of 10-20% over standard chow-fed rats, insulin resistance after as little as 2 weeks on a high fat diet, moderate hyperglycemia after prolonged exposure to high fat diet, elevated leptin levels, hepatic steatosis and changes to pancreatic β cells and insulin secretion.

Significant weight gain, hepatic steatosis, increased arterial wall thickness and a trend towards elevated leptin levels were observed in this study. The overall lack of increased insulin and hyperglycemia is surprising. However, there was increased variability in insulin levels, between animals, after 3 months on the diet. In mice it has been shown that 3 days is not long enough to increase insulin levels, but after 16-30 weeks on a high fat diet there is a change in basal insulin levels ^23, 24^. Random glucose tests and fasted glucose measures were taken, and it is evident that these rats did not display overt type 2 diabetes, unlike high-fat fed black/6 mice ^17, 25, 26^. It has been suggested that high-fat fed rats are not a reliable model of type 2 diabetes ^27^.

One possible explanation for the absence of these two key physiological changes could be the lack of systemic inflammation. Elevation of inflammatory markers and chronic low-grade systemic inflammation is regularly described in obese patients ^28, 29^, and inflammation in adipose tissue is thought to be linked to the development of insulin resistance and type 2 diabetes ^30, 31^. No changes in inflammatory markers were seen in this study. Changes in inflammatory markers are less consistently described in obese rodents but typically involve an increased concentration of circulating cytokines such as IL-6 ^24, 32^. This differs from the results found here, although it should be noted that most studies only provide a high fat diet for a brief period of time (days to weeks as opposed to months in the present study). Indeed, one study which reported increased circulating IL-6 after 3 days of diet consumption reported a non-significant reduction in this cytokine after 16 weeks ^24^, suggesting that changes in inflammatory markers may have been too transient and variable to be observed in this study.

The strain of rat used here (Wister-Han) may also be a contributor to the lack of diet-induced changes. Research has shown that genetic differences do affect the development of high fat diet -induced comorbidities such as insulin resistance ^33, 34^. Wister and Sprague-Dawley rats are more susceptible to high fat diet -induced changes than other rat strains ^11^. It is, therefore, possible that Wister-Han rats are a strain that is resistant to these changes. Additionally, within a strain some animals may be more resistant to diet induced obesity and metabolic syndrome than others ^35^.

It is evident that within this study this strain of rat did not develop the degree of comorbidities expected. There was no systemic increase in inflammatory markers, rise in blood pressure or significant changes in insulin or glucose levels. Additionally, the age at which the diet was started (12 months) and the length of time on the diet (8 months) may have reduced the comorbid outcomes. Inducing comorbidities is thought to be more effective when high fat diet consumption is started at a younger age ^11^, but in order to then use aged animals long term feeding of the high fat diet would be required. This may induce compensatory mechanisms, as in this study changes were evident in the first 2 months on the diet, some of which resolved by 8 months and others plateaued. To induce more robust changes, altering the type of diet fed may be of benefit. Increasing the fat content seems implausible as higher fat contents (above 60%) are rarely used and the physiological effects of these diets are unknown ^11^. Changing fat substance is also unlikely to help as lard (used in this study) has been shown to be more effective than plant-based fats such as olive oil or coconut oil ^36^. One feasible dietary change would be to use a high fat, high carbohydrate / sucrose diet which is commonly used as a model of type 2 diabetes in C57/BL6 mice ^37^. However, long term feeding of this diet (42 weeks) also shows some reversal of the comorbid state ^38^, compared to shorter durations on the diet (22 weeks). 

### Feasibility in stroke research

A second aim of this study was to determine whether the model developed could be used in stroke research by undertaking a number of procedures commonly used in the field. Despite the lack of comorbidities such as diabetes and hypertension in these rats, this work has clearly shown that aged, obese rats can be used in stroke research. These rats did not suffer from a high mortality rate as a direct result of stroke surgery and were able to undertake several tests for functional deficits. However, rats did need to be removed from the study due to age related complications ^4^ and 3 were removed due to non-stroke related surgical complications. The rats did continue to lose weight following stroke; however, due to their excessive size this was not a welfare concern. They also appeared to be more motivated in behavioural tasks following the stroke. For example, faster response times on the adhesive removal, which may have been in part due to the weight loss.

However, considerations do need to be made for testing these larger rats. Blinding during behavioural testing is not possible, due to the obvious size difference compared to control rats. The cylinder test did not work in this model, as they did not explore the cylinder, unlike younger counterparts. The aged rats also did not fit into restraining tubes for blood sampling, even the largest tubes on the market were not big enough. However, they were very placid to handle and restraint with a towel sufficed. One of the biggest issues encountered was the presence of heating issues during MCAO surgery. This was an unforeseen consequence of the large subcutaneous regions of adipose tissue, which not have a sufficient blood supply to disperse heat provided by a homoeothermic blanket during surgery, causing dermal burns. Monitoring of both internal and dermal temperature successfully counteracted this issue.

Due to the lack of expected comorbidities in these rats and other factors such as the low number of aged rats that reached the experimental end-point, it is difficult to make conclusions about the contribution of age and obesity to stroke outcomes from this feasibility pilot. Although from the limited evidence it appears there was little difference in infarct volume or behavioural outcome between the adult and aged rats. In the literature, aged animals (>18 months) have been shown to have reduced functional recovery after stroke ^39, 40^ while obese animals have been found to have increased infarct volume and functional deficits, particularly when fed a high fat diet for prolonged periods of time ^12, 13, 41^. Interestingly, not all studies using high-fat fed animals in stroke research have investigated the comorbidities present in their animals. The couple that have done so show the presence of diabetes but not hypertension in their animals and only compared obese and control animals up to 24hrs post-MCAO ^42, 43^. Other studies have used streptozotocin alongside a high fat diet to guarantee diabetes before demonstrating increased post-stroke deficits ^44, 45^. The lack of diabetes in this study could, therefore, be key to the lack of exacerbated post-stroke outcomes. Diabetes is a known risk factor for stroke and has been shown independently to increase post-stroke deficits ^4, 45^.

### Summary

The consumption of a high fat diet by aged Wister-Han rats did not cause the development of comorbidities typically seen in stroke patients, namely hypertension and diabetes. Therefore, these rats on a high fat diet alone are not an appropriate model for testing the combined effects of these comorbidities in stroke. There was a high degree of variability in many of the measures, adding to the already high logistical and financial costs of this model, reducing the feasibility of using this model in large drug development studies. Modifications to the methodology would be needed to ensure the development of desired comorbidities, in a reproducible fashion. However, this study does show that aged, obese rats can be used in stroke research, albeit with methodological modifications. There should be increased use of animals with comorbidities in pre-clinical stroke in order to adequately test the effects of novel interventions on stroke outcomes.

## Data availability

### Underlying data

Figshare: A pilot of the feasibility and usefulness of an aged obese model for use in stroke research. https://doi.org/10.6084/m9.figshare.12814949 ^22^.

This project contains the following underlying data:

-  rat weight pre MCO.csv (the weekly weights of the 11 aged obese rats included in the longitudinal analysis)
-  random glucose control.csv (the single timepoint random glucose measures collected from control rats as a benchmark)
-  random glucose high fat fed.csv (the monthly random glucose measures of the 11 aged obese rats included in the longitudinal analysis)
-  fasted glucose high fat fed.csv (the monthly fasted glucose measures collected from the 11 aged obese rats included in the longitudinal analysis)
-  Artery thickness.csv (the arterial thickness of thoracic and femoral arteries from aged obese and control animals)
-  Blood pressure control.csv (blood pressure measurements collected from control animals prior to MCAO surgery)
-  Blood pressure high fat fed.csv (blood pressure measurements collected from aged obese rats prior to MCAO surgery)
-  Weight loss post-MCAO as percentage of original weight.csv (percentage weight loss from pre-MCAO weight over 21 days post-MCAO for aged obese and control animals)
-  liver vacuole density.csv (the number of liver vacuoles /mm ^2^ in samples taken from aged obese and control animals)
-  Infarct volume.csv (Brain infarct volume as mm ^3^ for aged obese and control animals who underwent MCAO surgery)
-  Inflammatory markers high fat fed.csv (Monthly concentrations of 11 cytokines measured in aged obese rats over 8 months)
-  Metabolic measures High fat diet.csv (Monthly concentrations of 3 metabolic markers measured in aged obese rats over 8 months)
-  fig 2 statistics.pzfx (the GraphPad Prism file used to analyse data included in figure 2)
-  fig 3 statistics.pzfx (the GraphPad Prism file used to analyse data included in figure 3)
-  fig 4 statistics.pzfx (the GraphPad Prism file used to analyse data included in figure 4)
-  fig 5 statistics.pzfx (the GraphPad Prism file used to analyse data included in figure 5)

Data are available under the terms of the Creative Commons Zero "No rights reserved" data waiver (CC0 1.0 Public domain dedication).
