# Supplementary material for: A pilot of the feasibility and usefulness of an aged obese model for use in stroke research
Source: Wellcome Open Res. 2021 May 10;6:104. [Version 1] doi: 10.12688/wellcomeopenres.16592.1 (PMC8167504; doi:10.12688/wellcomeopenres.16592.1)
Supplement: Peer review history

# Peer review: Reviewer response for version 1

**DOI:** 10.21956/wellcomeopenres.18286.r43817

**Published:** 2021-06-03

**Authors:** Hilary Carswell

**Affiliations:** 1 Strathclyde Institute of Pharmacy and Biomedical Sciences, University of Strathclyde, Strathclyde, UK

This is a much needed pilot studying to establish the feasibility of MCAO in aged (20mths) and obese rats (60% fat diet for 8 months from 12months to mimic a poor diet in adulthood), multimorbidities associated with stroke incidence and severity. This is thorough and transparent, adhering to ARRIVE guidelines where possible and justified where not possible.

**Major comments:**

 Only male rats are used which could be seen as an additional risk factor for stroke, but certainly, it should be justified in the manuscript why only one sex was used and why were males chosen? (Please see: ‘Putting gender on the agenda’. Nature. 2010 Jun 10;465(7299):665. doi: 10.1038/465665a) ^1^

 Given this is a pilot study, it is essential that the authors perform power calculations, based on their pilot results, to inform future researchers on group sizes needed and to increase the impact of this study. 

 Justification for the doccol filament diameter is required – especially given different body weights in adult and aged animals

 Why not use rats with hypertension, diabetes or cholesterolemia if those are the multimorbidities you are targeting? 

 Comparing aged/obese with adult/non-obese complicates the interpretation of the data given two variables have changes and call for additional control groups (aged/non-obese; obese/adult…) 

 There is over 60% exclusion in aged/obese rats – this needs to be added to the results and conclusion as it has a bearing on whether these studies are really feasible.

**Minor comments:**

 What is meant by ‘substantial obesity’ in the abstract?

 In the abstract, please substantiate why ‘this model is not ideal for recapitulating the complex’ in the results so that the reader is better informed. 

 Need results of MCAO data in the abstract (survival, weight and functional outcome)

 The name of the pain relief, as well as frequency of administration in all animals, should be included as some have anti-inflammatory effects.

 It would be helpful to the reader to translate 65 years in humans to rats to justify the age of rats used (and/or 20 rat months to human years) (e.g. http://www.ratbehavior.org/RatYears.htm). Detailing 8 months of high fat diet in human years would also be helpful.

 Please include the infarct sizes and topography of lesions for the n=3 that underwent pilot MCAO for 20min or 30min. This seems to be missing and would help readers base their decision of occlusion time.

 Topography of the lesion might be different among aged/obese versus adult/non-obese so it would be helpful to see these.

 Details of the laser Doppler drop would be helpful especially if this was used as criteria for successful occlusion and would help readers establish this model but also because the drop may be different between aged/obese and adult/non-obese. 

 Were the animals still on high fat diet for the 4 weeks after MCAO? Please clarify

 Serum samples must have been frozen presumably so that 8month high fat diet serum samples could be analysed to inform on what inflammatory markers to measure at the earlier time points unless I’m missing something? The results of the 23 inflammatory markers at 8 months need to be included. How were the 11 markers then chosen given that only one time point for the 23 markers was used hence no compassions were made? 

 Lack of rearing in aged rats makes the cylinder test unfeasible – if this is new data, please state so. If not new data please put into context with other literature. 

 Despite the authors not analysing statistically the post-stroke data (infarct volume, histological measures and behaviour) because ‘the study was not designed or powered for these comparisons’ – post-MCAO body weight was statistically analysed (please clarify what the asterisks compare - with control or time (fig 5a)).

 Please indicate what the arrows are pointing to in fig 4a

 In the discussion, the authors comment on the aged/obese rats being ‘more motivated in behavioural tasks following the stroke. For example, faster response times on the adhesive removal, which may have been in part due to the weight loss’. I don’t see this faster response time in the data plus the inactivity or lack of rearing in the aged/obese rats in the cylinder test would indicate the opposite. Please clarify.

 The discussion mentions ‘methodological modifications’ needed for MCAO in aged obese rats- please detail what these are, as other than 20 or 30min occlusion time, these aren’t clear.

Is the work clearly and accurately presented and does it cite the current literature?

Yes

If applicable, is the statistical analysis and its interpretation appropriate?

Yes

Are all the source data underlying the results available to ensure full reproducibility?

Yes

Is the study design appropriate and is the work technically sound?

Yes

Are the conclusions drawn adequately supported by the results?

Yes

Are sufficient details of methods and analysis provided to allow replication by others?

Yes

Reviewer Expertise:

Preclinical stroke, brain damage and repair, stem cells, microglia, biomaterials.

I confirm that I have read this submission and believe that I have an appropriate level of expertise to confirm that it is of an acceptable scientific standard.

## References

[1] Putting gender on the agenda. *Nature* .2010;465(7299) : 10.1038/465665a 665 10.1038/465665a 20535156

# Peer review: Reviewer response for version 1

**DOI:** 10.21956/wellcomeopenres.18286.r43813

**Published:** 2021-05-28

**Authors:** Carles Justicia Mercader, Angélica Salas Perdomo

**Affiliations:** 1 Department of Brain Ischemia and Neurodegeneration, Institut d'Investigacions Biomèdiques de Barcelona (IIBB), Consejo Superior de Investigaciones Científicas (CSIC), Barcelona, Spain

In this work, the authors introduce several risk factors, such as aging and obesity, to the experimental stroke model. For this purpose, they used rats of advanced age and fed them a high-fat diet for 8 months, after which MCAo was performed. Various parameters have been measured (weight gain, blood pressure, inflammation markers, among others), as well as functional outcome after stroke induction.

 It is well known to researchers in the field of experimental stroke, that the vast majority of results obtained under "standard conditions" animal models (i.e. young healthy male rats) do not use to have a direct translation into clinical practice. This is due, in part, to the lack of adding risk factors that drive experimental models towards something closer to reality. From this perspective, the approach of the present manuscript makes a lot of sense. The results provided show that the experimental stroke model can be applied to aged and obese rodents, but on the other hand, that the high-fat diet in its experimental design has not produced the expected effects with obesity.

 Some questions arise that are worth commenting on:

 For a better analysis of results, it would be convenient to have corresponding controls for the two factors (aging and obesity), i.e. to add groups of aged rats on a standard diet and young rats on fat diet. Otherwise, we cannot find out how each of the factors influences the results.

 The chosen diet seems to have no effect on other parameters, such as hypertension, diabetes, or markers of inflammation. Perhaps other elements should be added to the diet that favors it. In fact, there is controversy as to whether obesity “per se” modifies the outcome of stroke, or whether it favors the appearance of other comorbidities such as diabetes, metabolic syndrome, or hypertension, among others.

 We would like more information on certain technical issues:

 Mention should be made of the exclusion criteria regarding blood flow drop during occlusion. Related to this, regarding the nylon filament used, why was that size chosen? Doccol recommends that diameter in rats from 320 to 360g, but the rats in this study are about 600g.

 The methodology does not mention whether the young animals were also habituated for blood pressure measurement.

Is the work clearly and accurately presented and does it cite the current literature?

Yes

If applicable, is the statistical analysis and its interpretation appropriate?

Not applicable

Are all the source data underlying the results available to ensure full reproducibility?

Yes

Is the study design appropriate and is the work technically sound?

Yes

Are the conclusions drawn adequately supported by the results?

Yes

Are sufficient details of methods and analysis provided to allow replication by others?

Partly

Reviewer Expertise:

Stroke; Neurodegeneration; Neuroimaging.

We confirm that we have read this submission and believe that we have an appropriate level of expertise to confirm that it is of an acceptable scientific standard.

# Peer review: Reviewer response for version 1

**DOI:** 10.21956/wellcomeopenres.18286.r43815

**Published:** 2021-05-25

**Authors:** Susanne Wegener

**Affiliations:** 1 Department of Neurology, University Hospital Zurich, Zurich, Switzerland

In their study, the authors make an important attempt to improve our current models of stroke in rodents. Risk factors should be incorporated, in order to reflect the real clinical situation of stroke patients. Aged male Wistar rats were fed a high-fat diet for 8 months before being subjected to MCAO. Throughout the 8 months, weight, blood pressure and metabolic/inflammatory serum markers were monitored. Despite some considerations (such as problems with maintaining body temperature during procedures in very obese rats), it was possible to induce stroke and assess functional impairment in this model of obesity.

 Other than expected, despite inducing obesity, high-fat diet did not affect stroke severity. Blood pressure did not increase significantly, and biomarkers of cardiovascular risk did not go up. Hepatic steatosis and an increased arterial wall thickness were found to increase.

 This study was very thoroughly performed; and it is very important that these results are published, although they were different than expected.

 It seems that in order to reflect full cardiovascular risk in aged stroke patients, the model needs to be modified.

**Minor points:** some factors that may have affected results should be discussed in the manuscript:

1. Type of diet: the high-fat diet may not suffice; other types of diet have been shown to indeed increase blood pressure and affect stroke severity and overall cerebral perfusion, such as the HFSS “cafeteria diet” (also increased salt and sugar content) by Langdon *et al.*,2018, Vol. 38(10) 1727–1740. ^1^ Please discuss why you did not choose this diet.
2. Please address the fact that only male rats were used as a limitation in the manuscript. The effect of diets may be different in females.

Is the work clearly and accurately presented and does it cite the current literature?

Yes

If applicable, is the statistical analysis and its interpretation appropriate?

Yes

Are all the source data underlying the results available to ensure full reproducibility?

Yes

Is the study design appropriate and is the work technically sound?

Yes

Are the conclusions drawn adequately supported by the results?

Yes

Are sufficient details of methods and analysis provided to allow replication by others?

Yes

Reviewer Expertise:

stroke (clinical and experimental)

I confirm that I have read this submission and believe that I have an appropriate level of expertise to confirm that it is of an acceptable scientific standard.

## References

[1] : Executive dysfunction and blockage of brain microvessels in a rat model of vascular cognitive impairment. *J Cereb Blood Flow Metab* .38(10) : 10.1177/0271678X17739219 1727-1740 10.1177/0271678X17739219 29083274PMC6168916
